# Supplementary material for: Excess forest mortality is consistently linked to drought across Europe
Source: Nat Commun. 2020 Dec 3;11:6200. doi: 10.1038/s41467-020-19924-1 (PMC7713373; doi:10.1038/s41467-020-19924-1)
Supplement: Supplementary file 2 — Supplementary Information [file 41467_2020_19924_MOESM2_ESM.pdf]

Supplementary Information

Excess forest canopy mortality is consistently linked to drought across Europe

Cornelius Senf, Allan Buras, Christian S. Zang, Anja Rammig, and Rupert Seidl

Table 1: Comparison of a null model against a model including CWB as predictor and a model including CWB as predictor and a smoothing term. Models were compared using the leave-one-out validated expected log predictive density (LOO-ELPD), which is a relative measure of predictive performance taking into account full model uncertainty. More positive LOO-ELPD values indicate higher predictive performance. We found the best predictive performance for the model including CWB as predictor and an additional smoothing term, allowing for a non-linear relationship between CWB and canopy mortality.

Model	ΔLOO-ELPD to best model (95 % credible interval)
CWB + smooth term	0 (0 – 0)
CWB	-221 (-264 – -177)
Null	-1614 (-1732 – -1498)

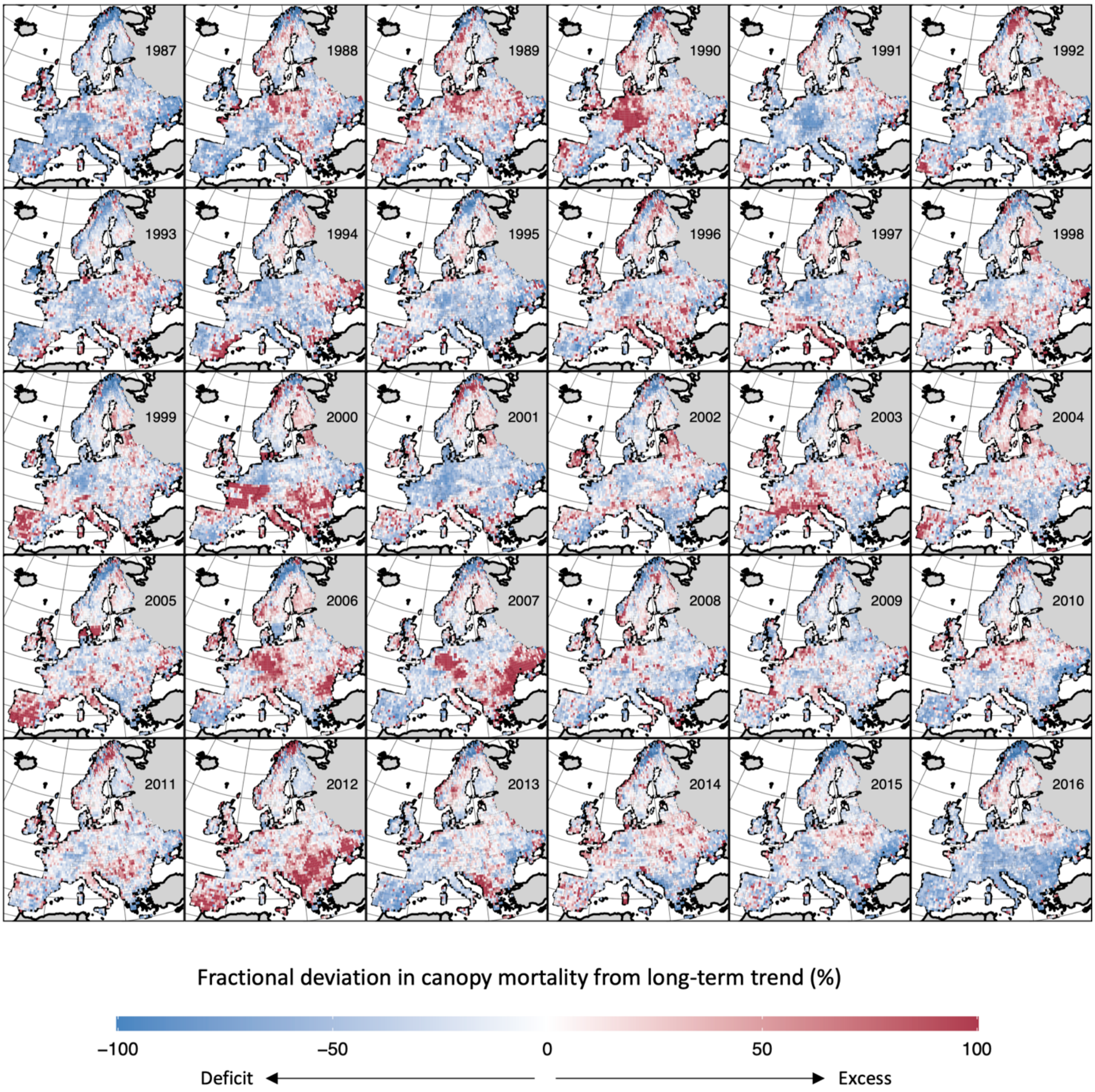

Figure 1: Fractional deviation in canopy mortality mapped across Europe over the period 1987 to 2016. Background maps are from https://naturalearthdata.com. The map was created by C. Senf.

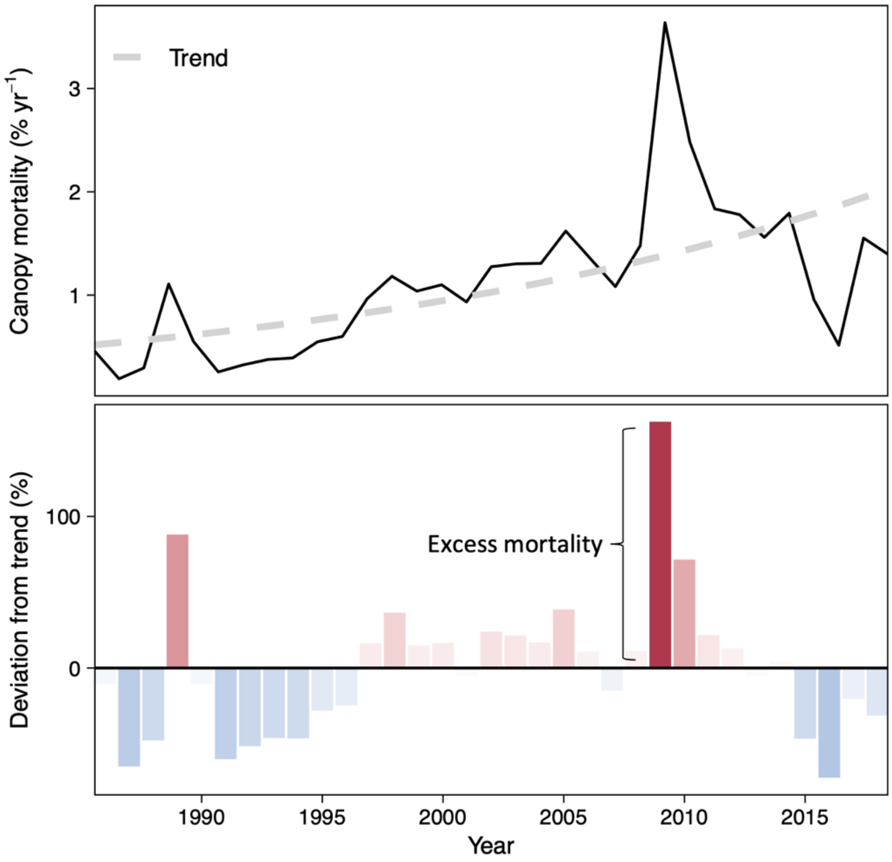

Figure 2: Visualization of the definition of excess forest canopy mortality as deviation from the long-term trend. The upper panel exemplarily shows the annual canopy mortality rate for one 0.5° grid cell (black line), with the trend line added (grey dashed line). The lower panel shows the deviation of the observed canopy mortality rate from the trend line, expressed as percent change from the fitted trend value. Positive deviation (i.e., reddish colors) indicate excess forest canopy mortality compared to the long-term trend.

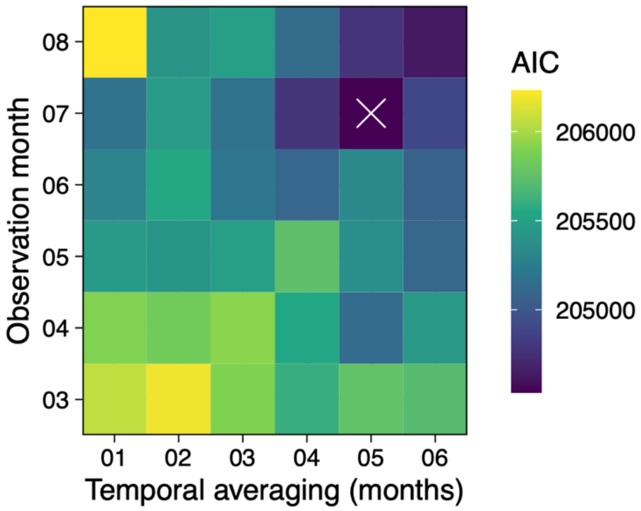

Figure 3: Model performance, measured via the Akaike Information Criteria, for different combinations of observation month and temporal averaging. The best combination is highlighted by a white cross. The window for temporal averaging is left-centered on the observation month, meaning that the best combination includes data from March to July.

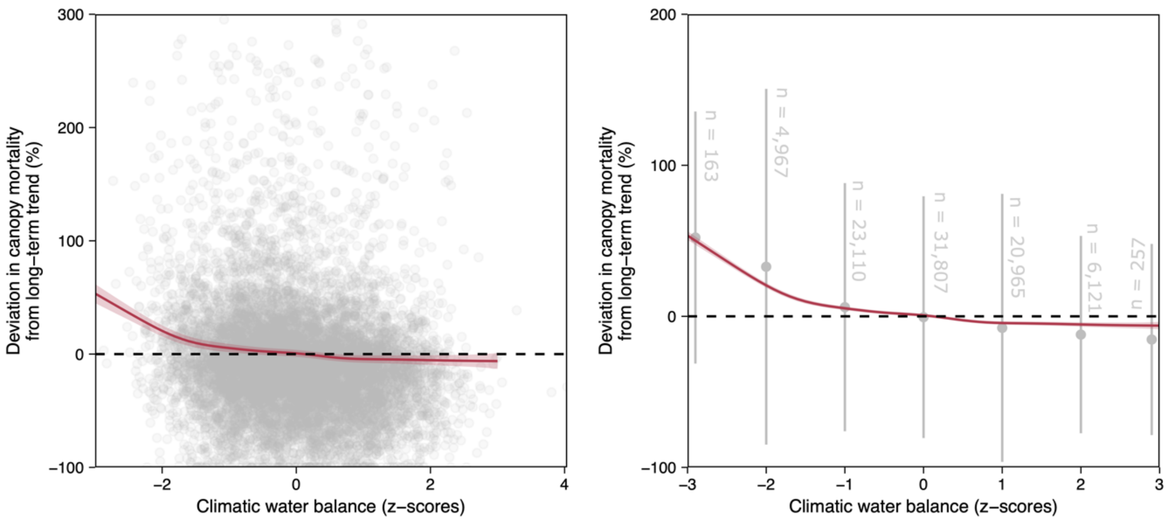

Figure 4: Expected (red line; derived from the best model in Table 1) and observed deviation in canopy mortality from the long-term trend (grey dots in left plots; aggregated to mean ± standard deviation in right plot) across n = 2,913 grid cells distributed equally across Europe (see Supplementary Figure 1). While there is high variability in the data, there is a clear non-linear relationship between CWB and the mean deviance in canopy mortality. That is, while a -1 standard deviation (SD) decrease in CWB leads to an average fractional increase in canopy mortality by 5.3 (1.8 – 8.9) %, canopy mortality increased on average by 20.5 (15.6 – 25.4) % with a -2 SD change in CWB, and by 53.5 (45.0 – 61.8) % with a -3 SD change in CWB. CWB explained approximately 6 % of the overall variability in mortality.

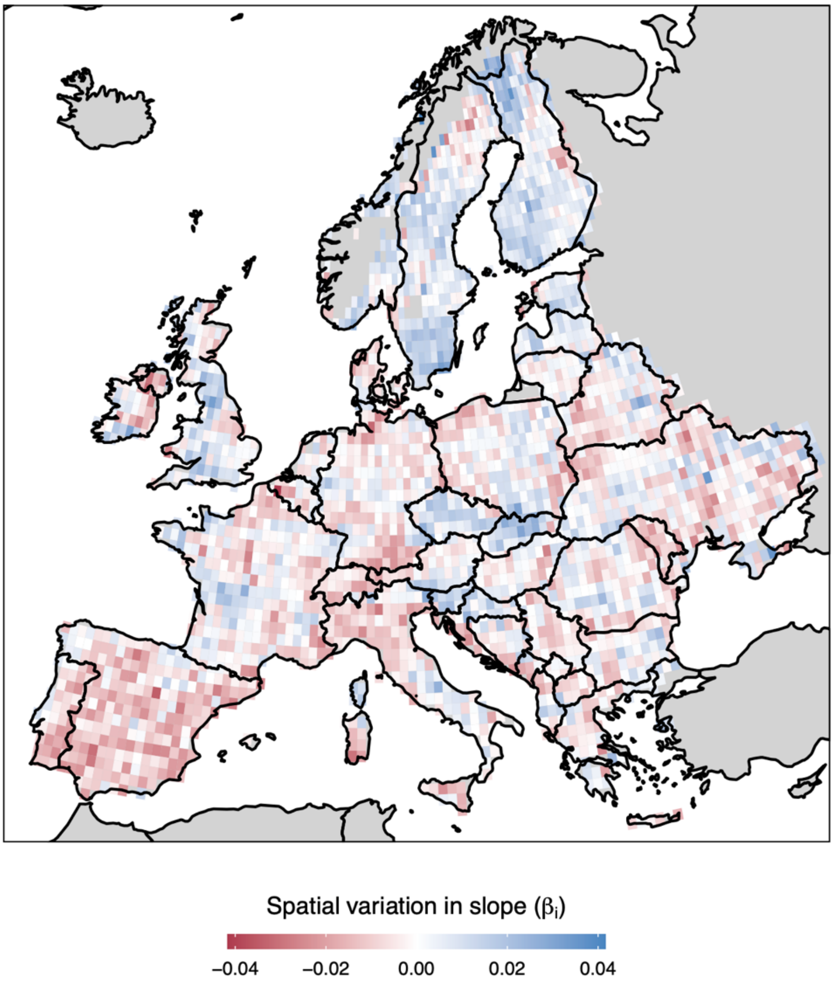

Figure 5: Spatial variability in regression slope (β_i) among grid cells. Red colors indicate that the grid cell had a stronger effect of CWB on variation in canopy mortality compared to the continental average (Table 2). Background maps are from https://naturalearthdata.com. The map was created by C. Senf.

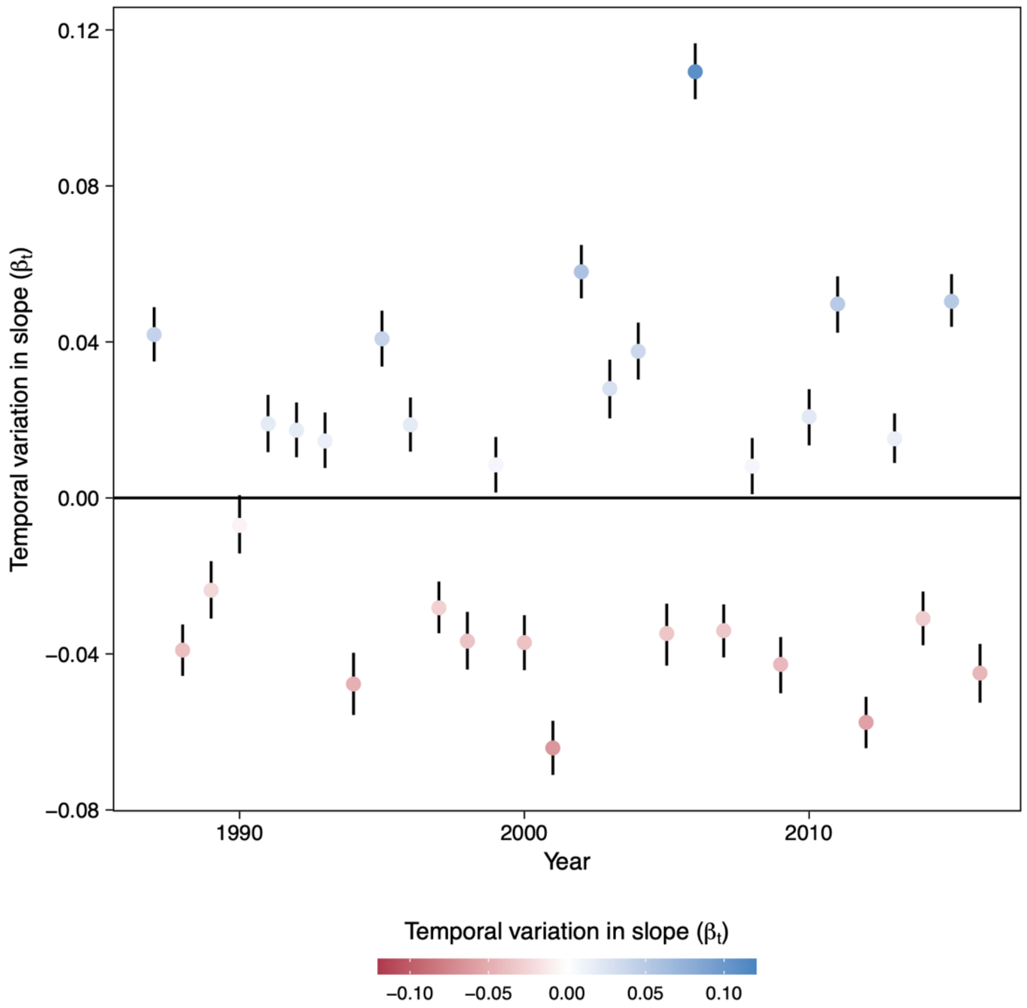

Figure 6: Temporal variability in regression slope (β_t) among years. Red colors indicate that during this year the effect of CWB on variation in canopy mortality was stronger compared to the temporal average (Table 2). Shown are the median and 95 % credible interval derived from all posterior draws (n = 8,000).

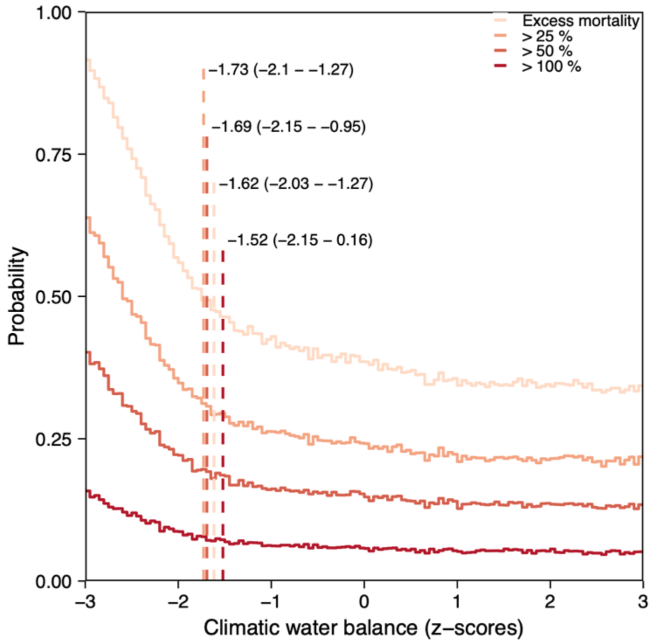

Figure 7: Thresholds values (and 95 % credible interval derived from n = 8,000 posterior draws) identified in the probability of excess canopy mortality over CWB.

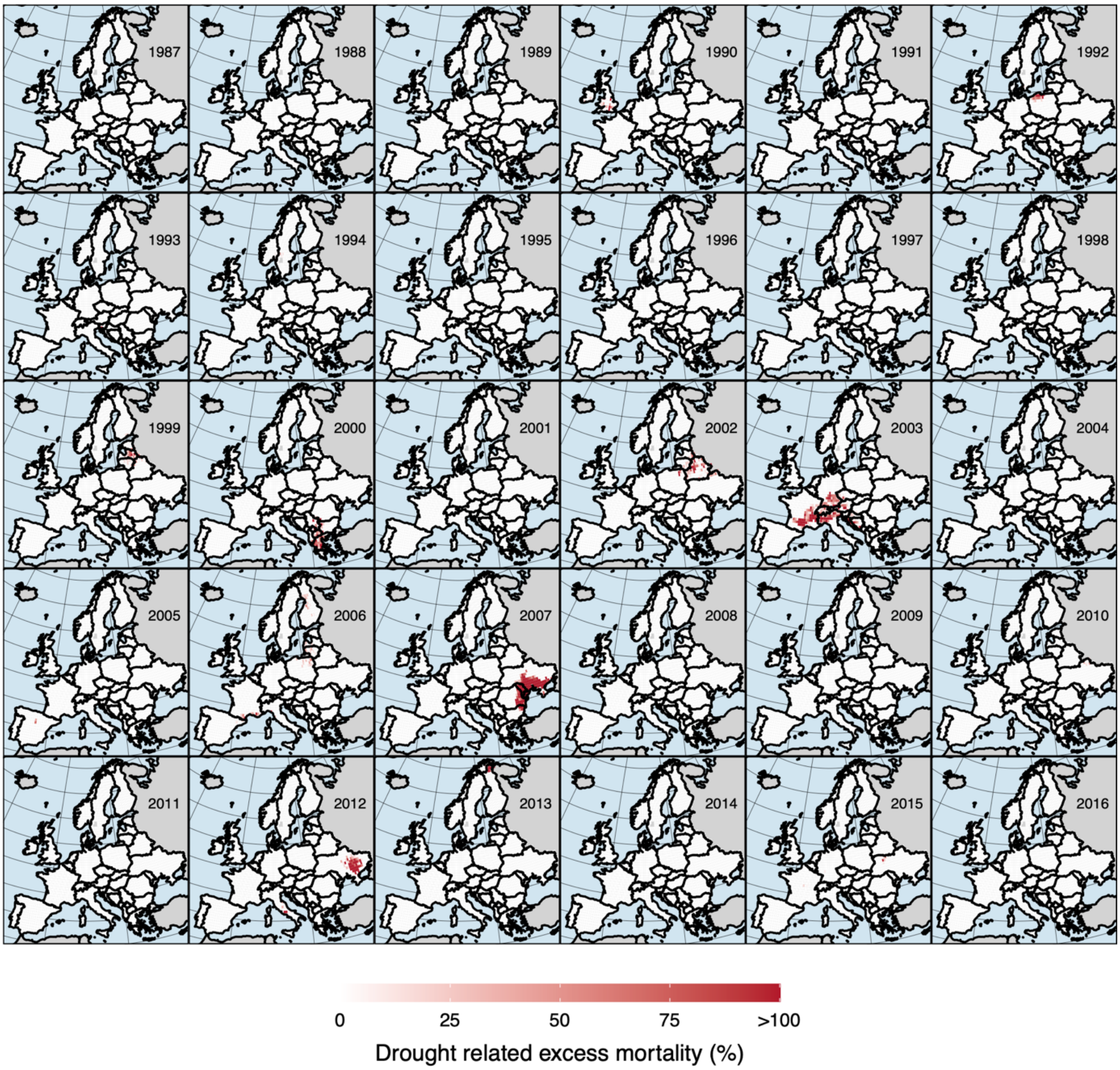

Figure 8: Same figure as Figure 2 but for the lower bound of the threshold. That is, hotspots are here defined as regions and years where excess canopy mortality coincided with CWB values < -2.0 standard deviations of the local average. Background maps are from https://naturalearthdata.com. The map was created by C. Senf.

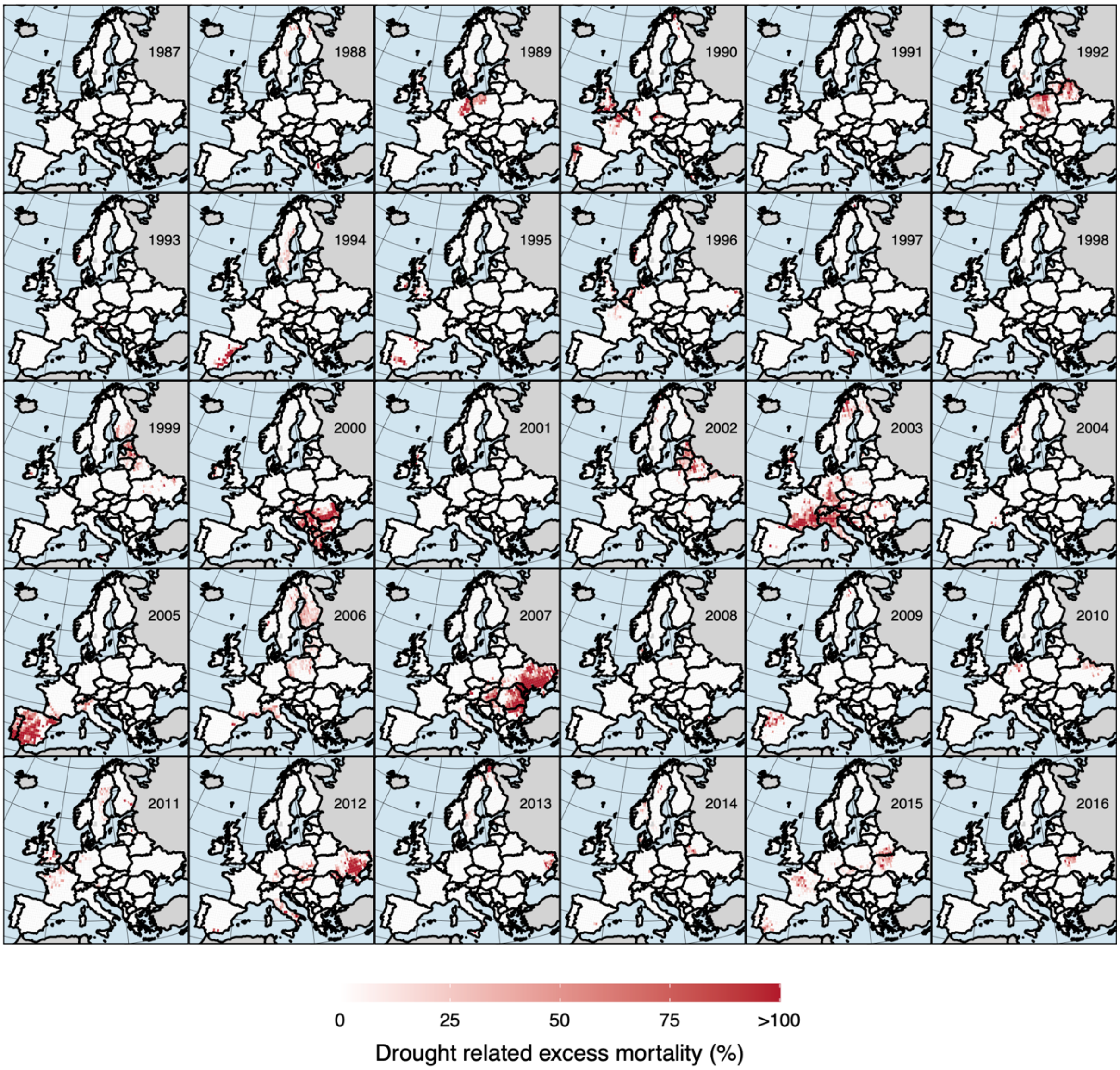

Figure 9: Same figure as Figure 2 but for the upper bound of the threshold. That is, hotspots are here defined as regions and years where excess canopy mortality coincided with CWB values < -1.3 standard deviations of the local average. Background maps are from https://naturalearthdata.com. The map was created by C. Senf.

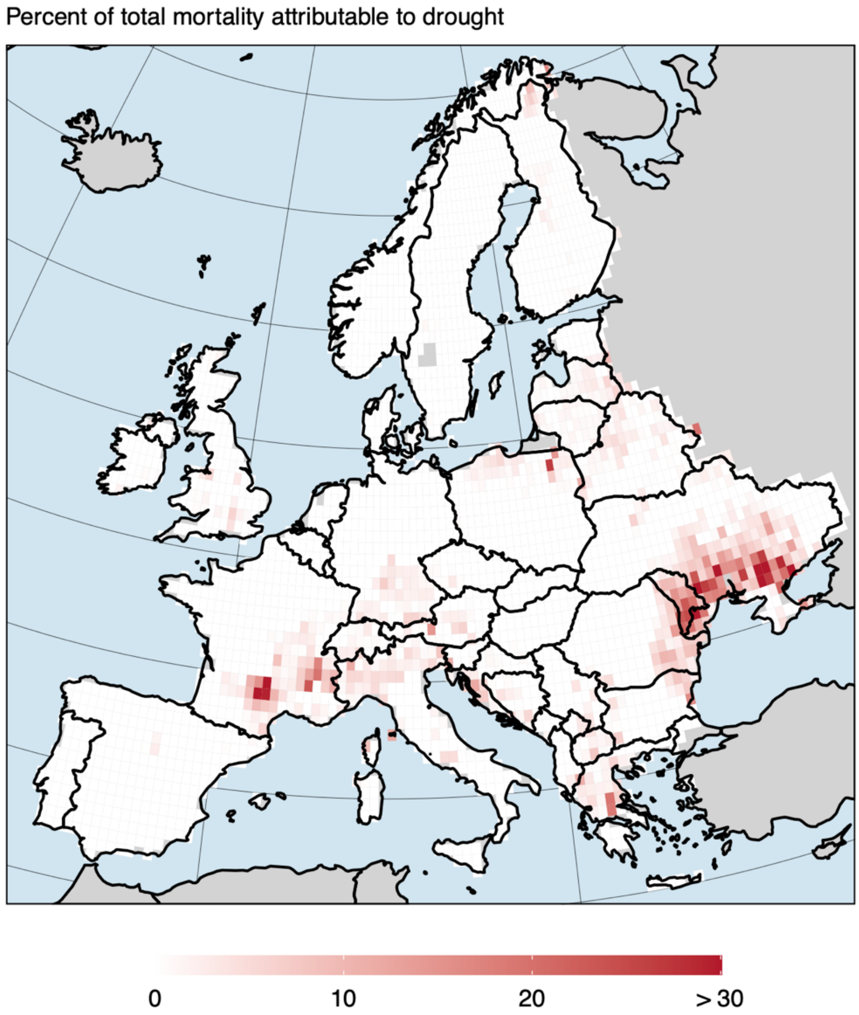

Figure 10: Same figure as Figure 4 but for the lower bound of the threshold. Background maps are from https://naturalearthdata.com. The map was created by C. Senf.

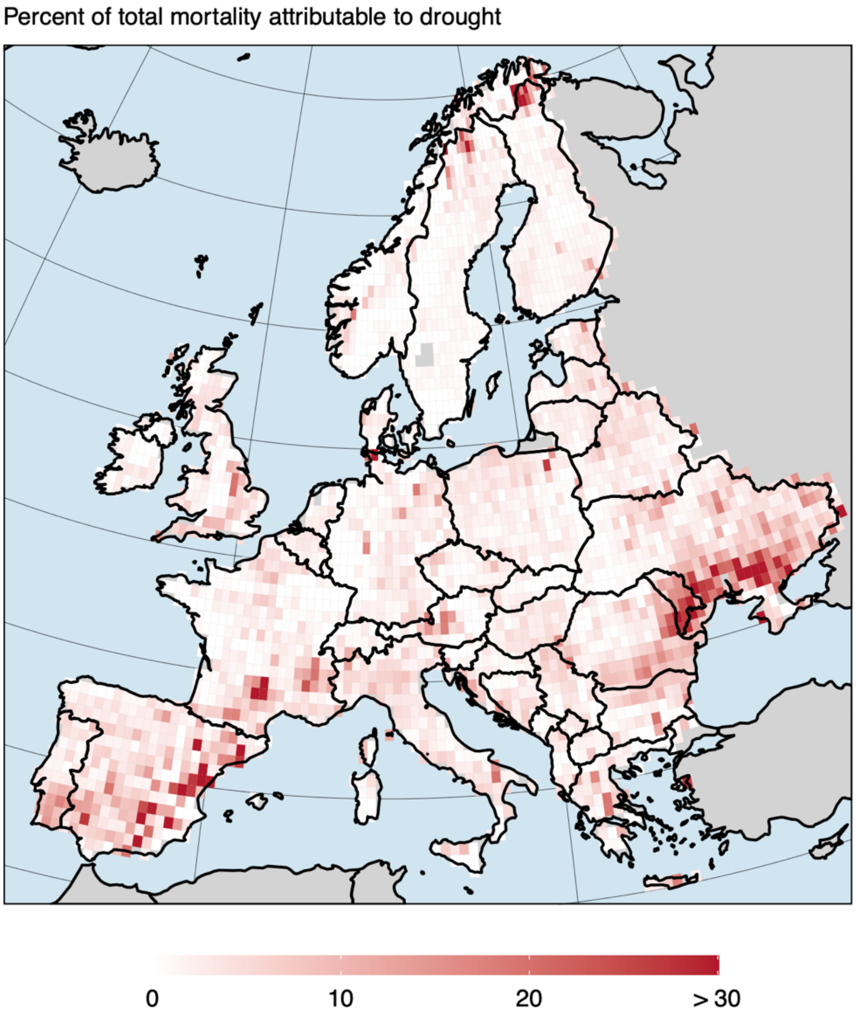

Figure 11: Same figure as Figure 4 but for the upper bound of the threshold. Background maps are from https://naturalearthdata.com. The map was created by C. Senf.

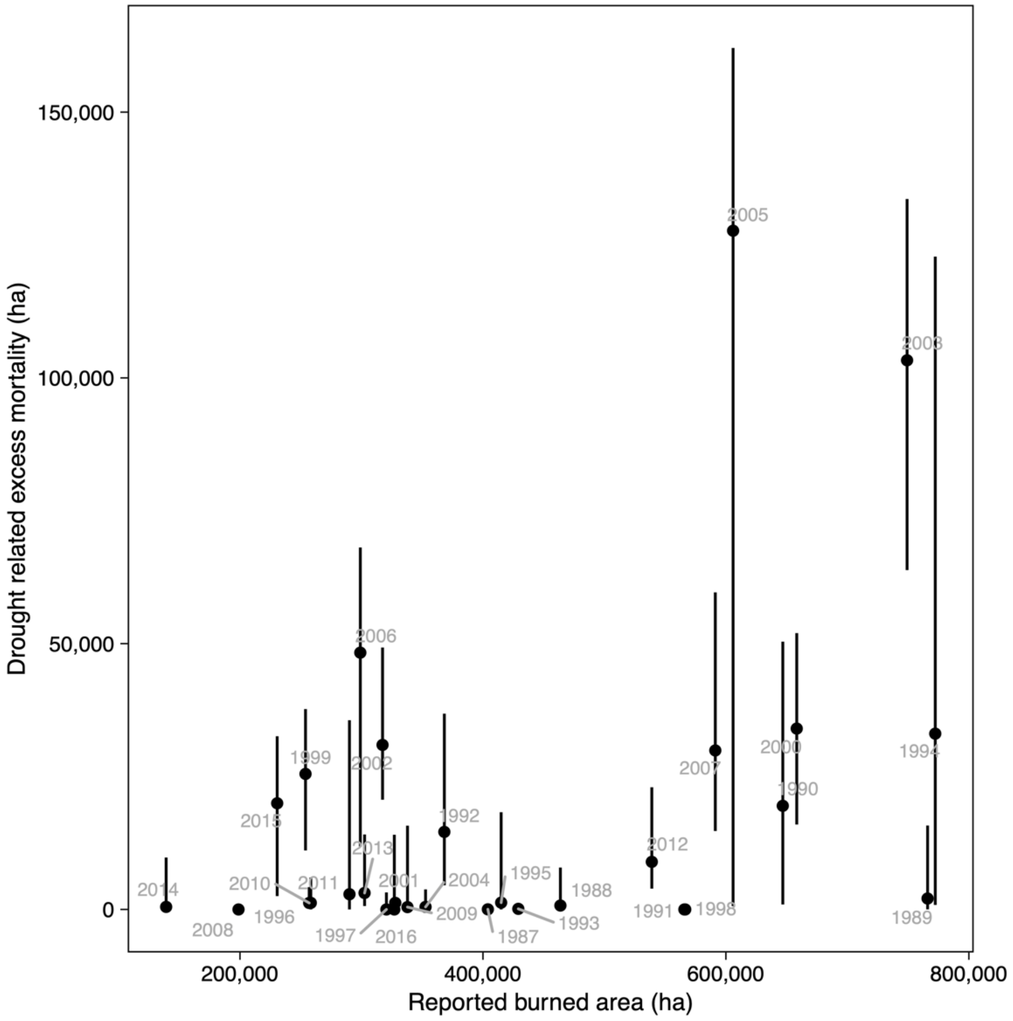

Figure 12: Self-reported burned area by European Union member states versus drought-related excess mortality reported in this study. Self-reported burned area was extracted from https://www.eea.europa.eu/data-and-maps/daviz/burnt-forest-area-in-five-2#tab-chart_4 (last accessed 17th of November 2020). Black dots indicate the area estimate using the average threshold for defining drought, whereas the error bars extent to the lower and upper uncertainty bound as reported in Figure 3 and Supplementary Figure 8 and Supplementary Figure 9.
